# Curcumin Induces Apoptosis of Upper Aerodigestive Tract Cancer Cells by Targeting Multiple Pathways

**DOI:** 10.1371/journal.pone.0124218

**Published:** 2015-04-24

**Authors:** A. R. M. Ruhul Amin, Abedul Haque, Mohammad Aminur Rahman, Zhuo Georgia Chen, Fadlo Raja Khuri, Dong Moon Shin

**Affiliations:** Department of Hematology and Medical Oncology, Winship Cancer Institute of Emory University, Atlanta, GA, 30322, United States of America; Duke University Medical Center, UNITED STATES

## Abstract

Curcumin, a natural compound isolated from the Indian spice "Haldi" or "curry powder", has been used for centuries as a traditional remedy for many ailments. Recently, the potential use of curcumin in cancer prevention and therapy urges studies to uncover the molecular mechanisms associated with its anti-tumor effects. In the current manuscript, we investigated the mechanism of curcumin-induced apoptosis in upper aerodigestive tract cancer cell lines and showed that curcumin-induced apoptosis is mediated by the modulation of multiple pathways such as induction of p73, and inhibition of p-AKT and Bcl-2. Treatment of cells with curcumin induced both p53 and the related protein p73 in head and neck and lung cancer cell lines. Inactivation of p73 by dominant negative p73 significantly protected cells from curcumin-induced apoptosis, whereas ablation of p53 by shRNA had no effect. Curcumin treatment also strongly inhibited p-AKT and Bcl-2 and overexpression of constitutively active AKT or Bcl-2 significantly inhibited curcumin-induced apoptosis. Taken together, our findings suggest that curcumin-induced apoptosis is mediated via activating tumor suppressor p73 and inhibiting p-AKT and Bcl-2.

## Introduction

Cancer is the second leading cause of death in the United States and is projected to claim 2.3 million lives in 2014 [1]. Smoking or tobacco use is the cause of more than 30% of all cancers, which predominantly affect the upper aerodigestive tract including the lung and bronchus, larynx, pharynx and oral cavity. Squamous cell carcinoma of the head and neck (SCCHN) and lung cancers are the two major tobacco-related cancers. Tremendous advances have been made over last few decades in the field of cancer prevention and therapy and the number of cancer survivors has increased from 3 million in 1971 to 13.7 million in 2012 (AACR Cancer Progress Report, 2013). However, the safety of available drugs remains a major concern, since most currently used drugs are highly toxic. On the other hand, natural dietary compounds present in fruits, vegetables and spices have been used in traditional medicines over centuries for various therapeutic purposes and their safety has been established through human consumption over years. Hundreds of natural dietary compounds have been investigated for their anti-cancer effects in the last several decades [2, 3]. Unlike chemotherapy and molecularly targeted agents, the beauty of natural compounds is their safety and context-dependent effects on multiple signal transduction pathways [2]. This multi-targeted effect is desired for the prevention and therapy of multi-factorial diseases such as cancer, which involves complex interactions between multiple signal transduction pathways.

Among the thousands of natural compounds initially tested for their anti-cancer potential, only about 40 promising agents have been moved to clinical trials. Curcumin, a diketone compound isolated from the rhizomes of the plant *Curcuma longa* commonly known as “Haldi” in the Indian subcontinent, is one such agent currently under clinical investigation [4]. The anti-cancer potential of curcumin has been established through multiple animal studies. Curcumin was found to significantly decrease the initiation of 7,12-dimethylbenz-[a]-anthracene (DMBA)-induced mammary adenocarcinoma in female rats by its intraperitoneal infusion 4 days before DMBA administration [5]. On the other hand, the chemopreventive effect of curcumin on *N*-nitrosomethylbenzylamine-induced esophageal carcinogenesis in rats was observed not only in the initiation phase but also in post-initiation phases [6]. Curcumin was also reported to prevent colon cancer development in C57Bl/6J Min/+ mice with *APC* mutation [7] and *N*-nitrosodiethylamine and phenobarbital-induced hepatic cancer in rats and reduced lipid peroxidation and salvaged hepatic glutathione antioxidant defense [8]. The results of several clinical trials have also been published which showed curcumin to be a promising chemopreventive agent, and are summarized in [4]. Like many other natural compounds, curcumin modulates multiple signal transduction pathways involved in the lengthy carcinogenesis process and induces apoptosis, inhibits survival signals, scavenges reactive oxidative species (ROS), and reduces the inflammatory cancer microenvironment depending on the study context [4]. In the current study, we investigated the mechanism of curcumin-induced apoptosis in upper aerodigestive tract (lung and head and neck) cancer cell lines and showed that curcumin inhibited survival signals (p-AKT and Bcl-2), the reversal of which protected cells. On the other hand, curcumin activated tumor suppressor pathways such as p73, inactivation of which also protected cells from curcumin-induced apoptosis.

## Materials and Methods

### Cell lines

Cell lines used in the study were described elsewhere [9, 10]. Tu212, a cell line of hypopharyngeal origin, was kindly provided by Dr. Gary L. Clayman (University of Texas M.D. Anderson Cancer Center, Houston, TX). Tu686 from a primary tongue cancer and 886LN from the lymph node of laryngeal cancer origin were gifts from Dr. Peter G. Sacks (New York University College of Dentistry, New York, NY). The human lung cancer cell lines A549, H1299, H460 and H292 were obtained from Dr. Sun’s laboratory (Emory University). MDAH041 (041) is a human fibroblast cell line isolated from a Li-Fraumeni Syndrome patient and maintained in DMEM containing 10% FBS. The SCCHN cell lines were maintained in DMEM/F12 (1:1) medium supplemented with 10% heat-inactivated fetal bovine serum in a 37°C, 5% CO_2_ humidified incubator. Lung cancer cell lines were maintained in RPMI-1640 media supplemented with 5% FBS.

### Treatment of cells with curcumin

Curcumin was purchased from Sigma-Aldrich (St. Louis, MO) and was dissolved in DMSO as a stock solution, which was further diluted in cell culture media immediately before use. The final concentration of DMSO was <0.1%. All cells were plated at a concentration of 2.5X10^5^ cells/6-cm dish the day before treatment and treated with curcumin after overnight incubation.

### Measurement of IC_50_

Appropriate numbers of cells were seeded with 100 μL medium in 96 well culture plates and incubated overnight before treatment with varying concentrations of curcumin for 72 h. Inhibition of cell growth was determined by sulforhodamine B (SRB) assay as described elsewhere [11, 12]. IC_50_ values were calculated using CalcuSyn software (Biosoft, UK).

### Annexin V-phycoerythrin staining for apoptosis

Cells were treated with curcumin as indicated in the figure legends, trypsinized and washed in cold 1x PBS. The cells were then resuspended in 1x Annexin binding buffer (BD PharMingen, San Diego, CA), and stained with Annexin V-phycoerythrin (Annexin V-PE; BD PharMingen, San Diego, CA) and 7-AAD (BD PharMingen, San Diego, CA) for 15 min at room temperature. The stained samples were analyzed using a fluorescence-activated cell sorting caliber bench-top flow cytometer (BD Biosciences, San Jose, CA). Data were analyzed for the apoptotic population using FlowJo software (Tree Star, Ashland, OR).

### Western blot analysis

Cells were treated with curcumin for the indicated times and whole cell lysates were extracted using lysis buffer. 20–30 micrograms of protein was separated on 8–12% SDS-PAGE, transferred onto a polyvinylidene difluoride membrane (Millipore Corporation, Billerica, MA) and immunoblotted with specific antibodies. Mouse anti–β-actin antibody (Trevigen, Gaithersburg, MD) was used as a sample loading control. Immunostained protein bands were detected with an enhanced chemiluminescence kit (Thermo Scientific, Rockfield, IL).

### Generation of transfected cell lines

The dominant-negative p73 plasmid generation and validation is described elsewhere [13]. pWZL-NeoAKT plasmid (constitutively active) was obtained from Addgene, and pLNCX-Bcl-2 plasmid was obtained from the laboratory of Dr. M.W. Jackson (Case Western Reserve University, Cleveland, Ohio). The Bcl-2 overexpressing 041 cell line was generated by retroviral transduction of Bcl-2 and selection by G418 as described [14]. Constitutively active Akt was overexpressed in Tu686 cell lines (G418 selected pool).

## Results

### Curcumin inhibits growth and induces apoptosis of SCCHN and lung cancer cell lines

**Fig 1:**
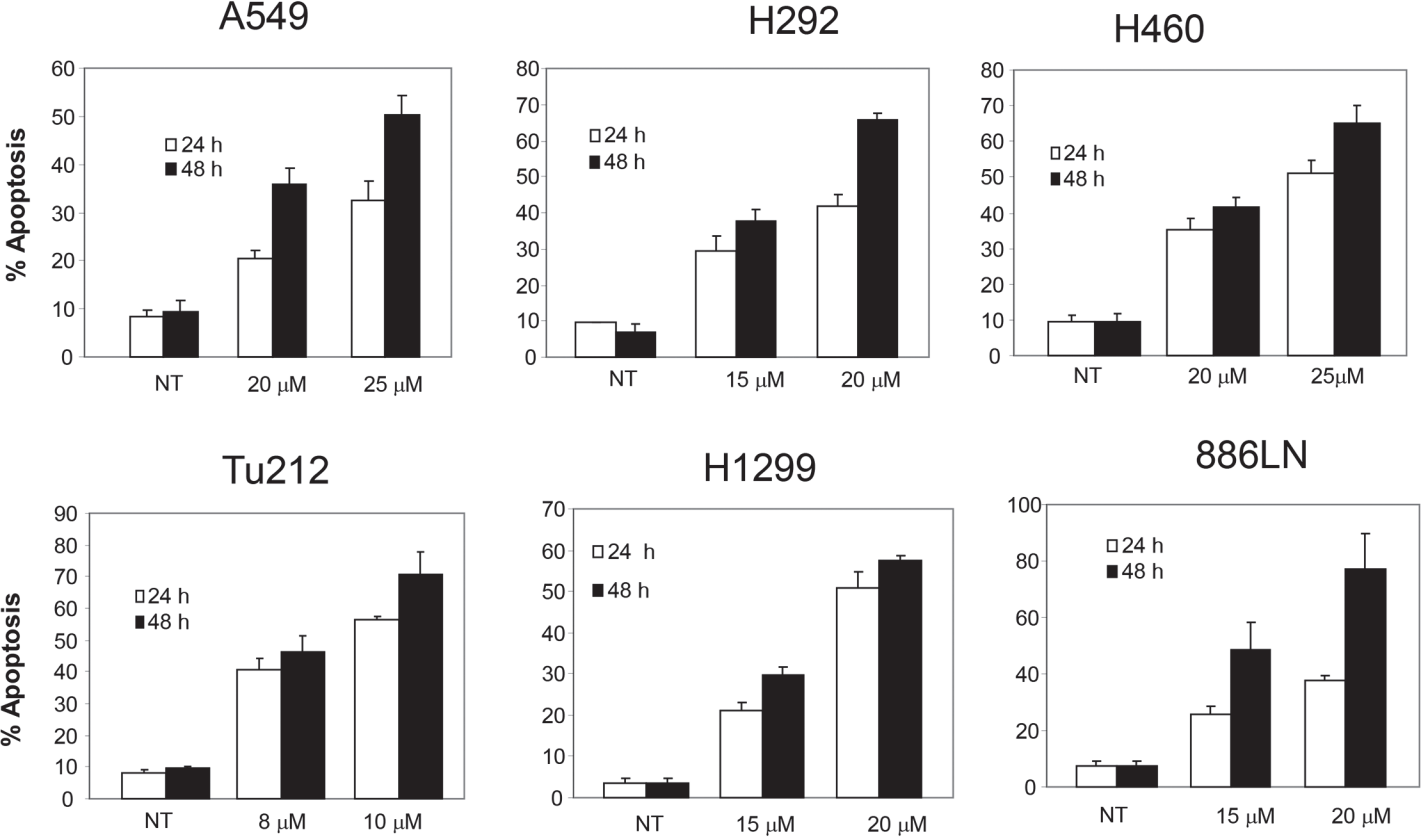
Curcumin dose- and time-dependently induces apoptosis of upper aerodigestive tract cancer cells. Lung cancer cell lines A549, H292, H460, H1299, and SCCHN cell lines Tu212 and 886LN were treated with the indicated concentration of curcumin for 24 and 48 h. Apoptosis was measured by annexin V-PE staining. Average apoptosis from three independent experiments is presented with standard deviation as error bars.

**Fig 2:**
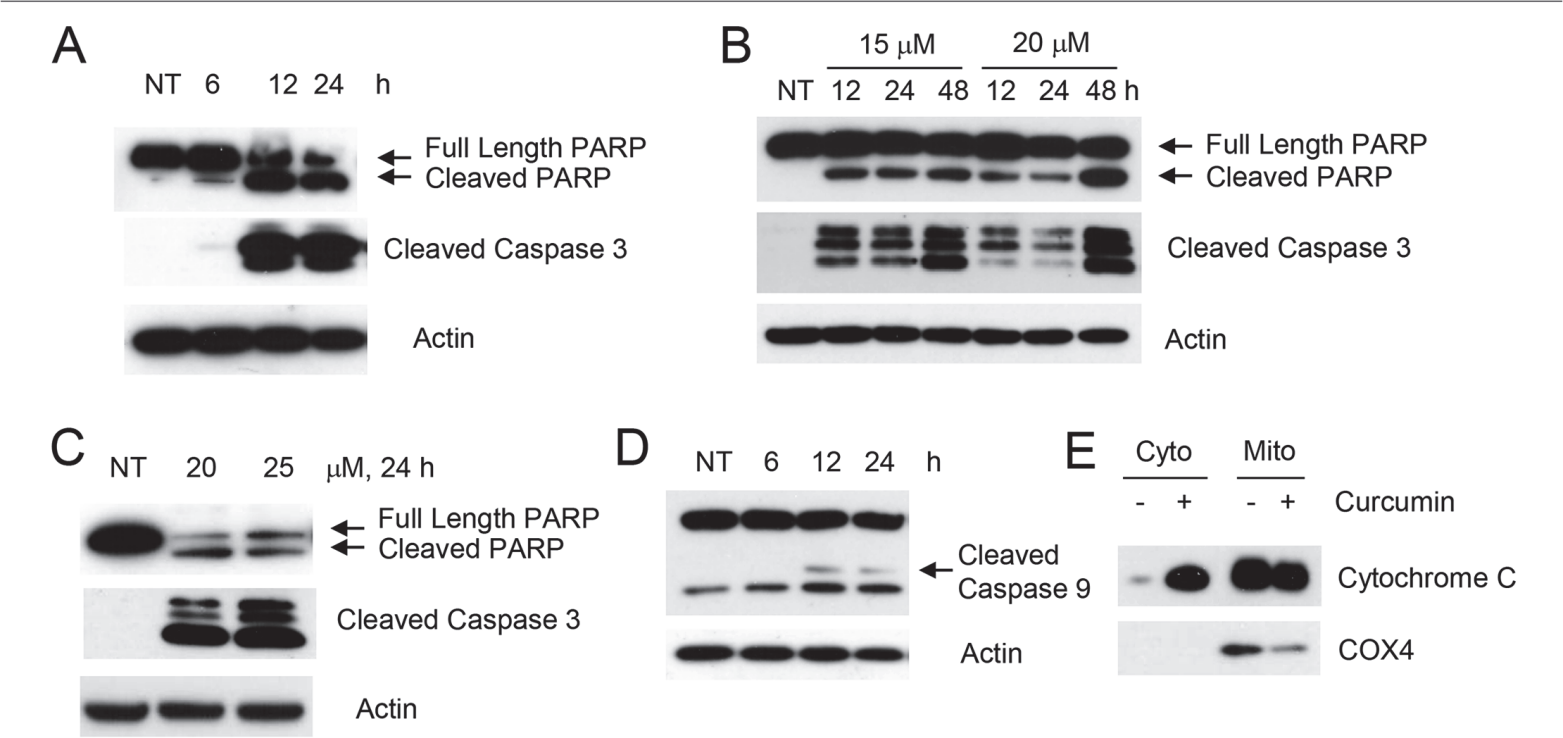
Curcumin induces mitochondria-mediated apoptosis. (A) Tu212, (B) H1299 and (C) H292 cells were treated with the indicated concentration of curcumin for the indicated times. Whole cell lysates were immunoblotted with PARP and caspase 3 (detects cleaved form only) antibodies. (D) Tu212 cells were treated with 10 μM of curcumin for the indicated times and whole cell lysates were blotted with caspase 9 antibody. (E) Tu212 cells were treated with 10 μM of curcumin for 24h. Cytoplasmic and mitochondrial fractions were separated and immunoblotted with cytochrome *c* antibody. COX4 (a mitochondrial protein) was used to show efficiency of cell fractionation. Representative data from three independent experiments are shown.

Curcumin has drawn particular attention because of its potential chemopreventive and anti-tumor effects and has been widely investigated from the late 1980s. However, the mechanism of its anti-cancer efficacy is not yet fully understood. More importantly, curcumin targets multiple signal transduction pathways which vary greatly depending on the tumor type. In order to understand the mechanism of curcumin-induced apoptosis in upper aerodigestive tract cancers, we first examined the sensitivity of multiple SCCHN cell lines to varying doses of curcumin. As shown in S1 Fig, all cell lines were sensitive to curcumin-induced growth inhibition although their degree of sensitivity varied. Tu212, Tu177 and 886LN cell lines were more sensitive than 38 and SqCCy1 cell lines. We also measured the IC50 values of curcumin in several SCCHN and lung cancer cell lines, which also suggested varying degree of sensitivities of these cell lines (Table 1). In general, SCCHN cell lines were more sensitive than lung cancer cell lines. We next measured apoptosis after curcumin treatment in different lung and SCCHN cell lines. As shown in Fig 1, curcumin dose- and time-dependently induced apoptosis of different cell lines. Although sensitivity varied among cell lines, 10–25 μM curcumin induced more than 50% apoptosis by 48 h. To further confirm apoptosis induction by curcumin, we examined cleavage of PARP and caspase 3 in three different cell lines (one SCCHN and two lung), which suggested that curcumin dose- and time-dependently cleaved PARP and caspase 3 in these cell lines (Fig 2A–2C). We next examined whether curcumin induced intrinsic or extrinsic apoptosis by measuring cytochrome c release and caspase 9 cleavage. As shown in Fig 2D and 2E, curcumin also cleaved caspase 9 and efficiently released cytochrome c in the cytoplasm, suggesting that curcumin induced mitochondrial-mediated intrinsic apoptosis.

**Table 1:** IC_50_ values of curcumin.

Cell Line	IC_50_ (μM)
A549	11.2
H1299	6.03
H292	11.6
Tu212	5.5
Tu686	6.4

### Curcumin induces p73-dependent but p53-independent apoptosis

**Fig 3:**
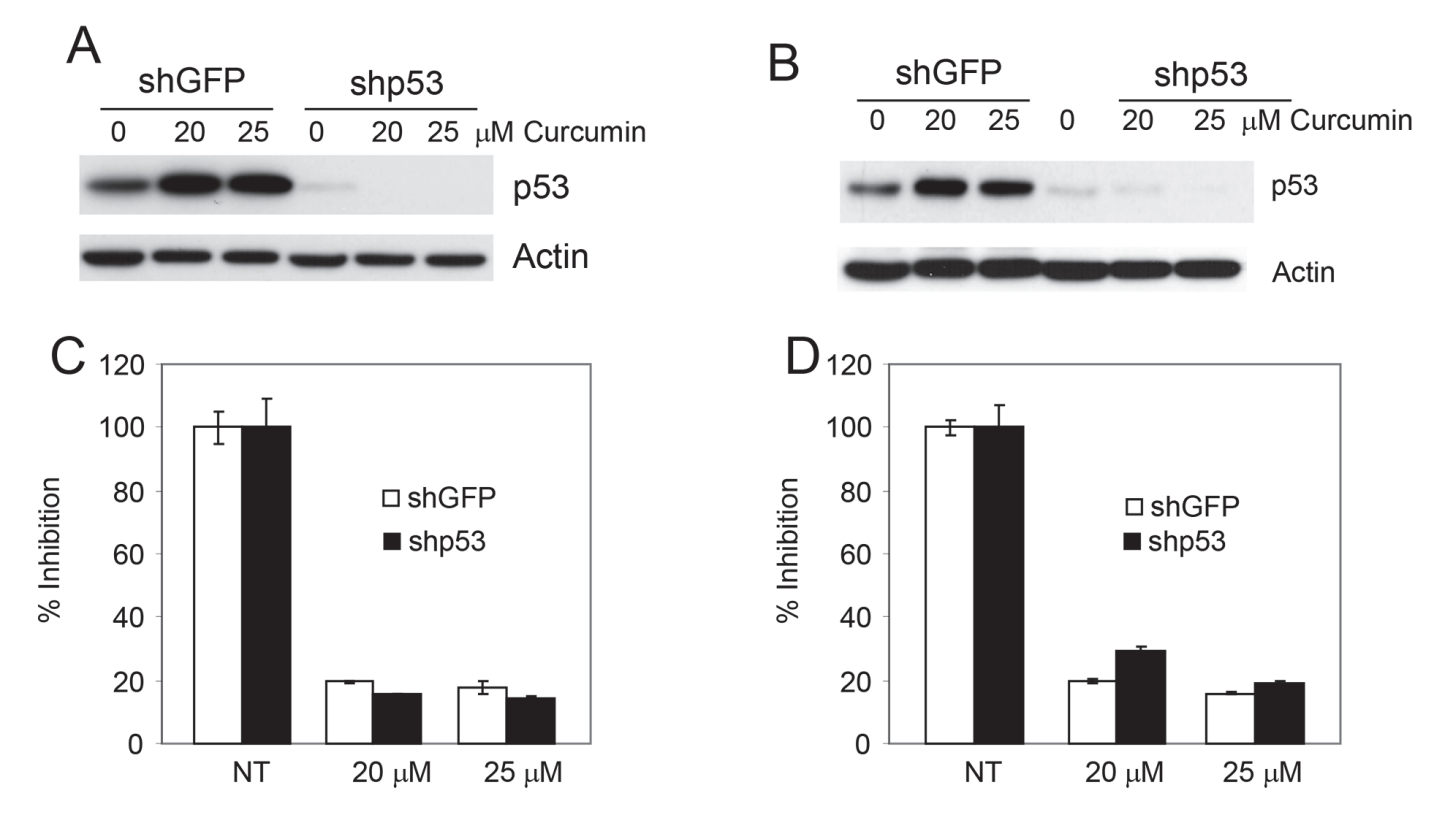
Curcumin induces p53-independent apoptosis. (A-B) The expression of p53 was ablated in H292 (A) and A549 (B) cells using shRNA as described in the Methods section and expression of p53 was measured by immunoblotting. (C) H292 and (D) A549 cells were treated with curcumin and cellular growth was measured by SRB assay.

**Fig 4:**
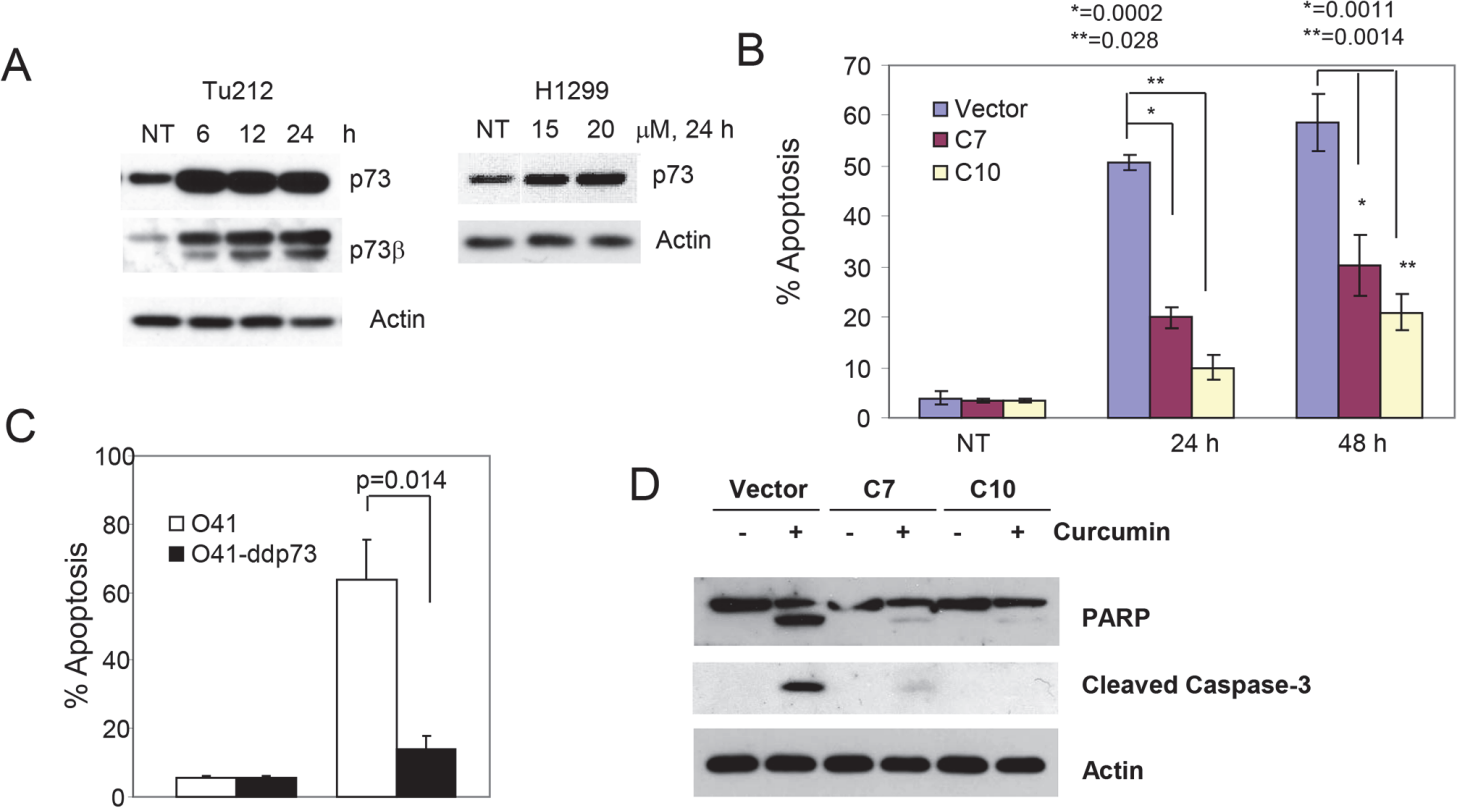
Curcumin induces p73-dependent apoptosis. (A) Tu212 (left) and H1299 (right) cells were treated with curcumin and expression of p73 and p73β were measured by Western blotting. (B-C) p73 was inactivated in H1299 (B) and 041 (C) cells by dominant negative p73 and apoptosis was measured by annexin V-PE staining. (D) H1299 cells expressing empty vector or dominant negative p73 were treated with curcumin. Whole cell lysates were immunoblotted with PARP and caspase 3 antibodies. For B and C, average results from three independent experiments were plotted with standard deviation as error bars. p values were determined by student t-test. p<0.05 was considered statistically significant.

The p53 family consists of three structurally and functionally closely related proteins, p53, p63 and p73, functionally classified as transcription factors and tumor suppressors, which play a critical role in apoptosis [15]. Although curcumin increased the expression of p53, the exact role of p53 in curcumin-induced apoptosis is not clear. Moreover, curcumin induced p53-independent apoptosis in lung cancer cell lines [16, 17] although the mechanism is not well understood. To investigate the role of p53 in curcumin-induced apoptosis, we used two pairs of isogenic lung cancer cell lines (H292 and A549) previously generated in our laboratory in which the parental cell lines express wild-type p53 which was ablated using shRNA [9]. As shown in Fig 3, treatment with curcumin increased the level of p53 in both cell lines. On the other hand, curcumin-induced growth inhibition was similar in both cell lines, suggesting that curcumin inhibited the growth of lung cancer cell lines in a p53-independent manner. As shown in Fig 1, curcumin-induced growth inhibition in these cell lines was due to apoptosis. We have previously reported that p73 also plays a critical role in drug (both natural and synthetic)-induced apoptosis [10, 13, 14]. Therefore, we examined the expression of p73 in two cell lines that express inactive p53 and found that curcumin increased the expression of p73, particularly the β-isoform, in both cell lines (Fig 4A). To confirm the role of p73 in curcumin-induced apoptosis, we inactivated p73 in H1299 [18] and 041 cells [10] by overexpressing dominant negative p73 and measured apoptosis after curcumin treatment. Curcumin-induced apoptosis was significantly inhibited after p73 inactivation (Fig 4B and 4C). p73-dependent apoptosis by curcumin was further confirmed by inhibition of PARP and caspase 3 cleavage (Fig 4D).

### Curcumin induces apoptosis by inhibiting AKT activation

**Fig 5:**
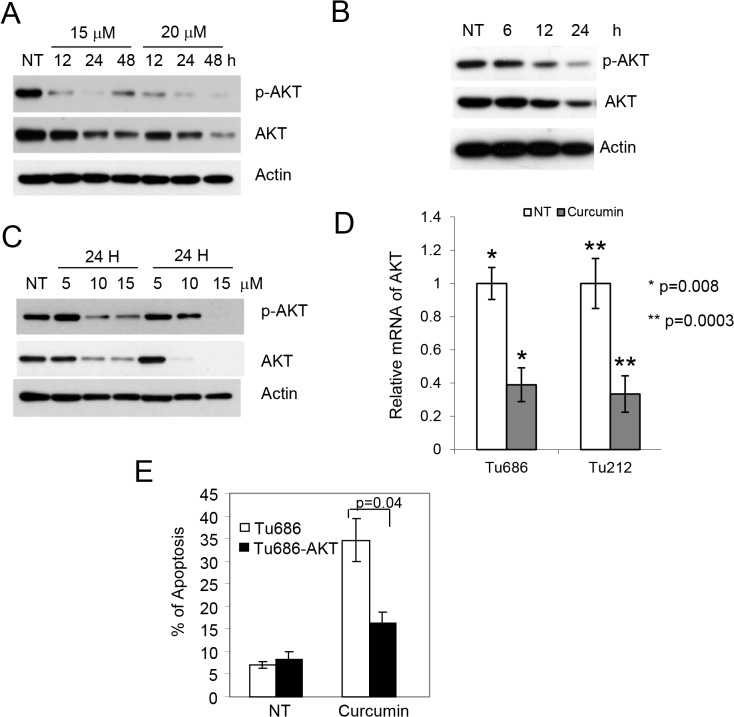
Inhibition of p-AKT is required for curcumin-induced apoptosis. (A) H1299, (B) Tu212 and (C) Tu686 cells were treated with the indicated concentrations of curcumin for the indicated time and expression of p-AKT, AKT and actin were measured by immunoblotting. (D) Tu212 and Tu686 cells were treated with 10 and 15 μM of curcumin, respectively. Expression of AKT mRNA was measured by qPCR. (E) CA-AKT was overexpressed in Tu686 cells and apoptosis was measured. Average results from three independent experiments were plotted with standard deviation as error bars. p value was determined by student t-test. p<0.05 was considered statistically significant.

One of the advantages of natural compounds over targeted agents is their multi-targeted effects. Most aerodigestive tract malignancies are initiated by oncogene activation, and the subsequent activation of AKT through phosphorylation by these oncogenes is a major and common downstream event. Therefore, we examined the p-AKT level after treatment with curcumin and found that curcumin treatment strongly inhibited the phosphorylation of AKT as well as the level of total AKT in all cell lines tested (Fig 5A–5C). We measured the mRNA expression of AKT after curcumin treatment. As shown in Fig 5D, curcumin also inhibited the level of AKT mRNA in all cell lines. To confirm that inhibition of p-AKT mediates apoptosis, we overexpressed constitutively active AKT (CA-AKT) in Tu686 cells and established a pool of cells expressing the plasmid by G418 selection as described [18]. These cells were treated with curcumin and apoptosis was measured. As shown in Fig 5E, overexpression of CA-AKT significantly protected cells from curcumin-induced apoptosis. These results suggest that curcumin-induced apoptosis is mediated via inhibition of p-AKT.

### Role of inhibition of Bcl-2 expression in curcumin-induced apoptosis

**Fig 6:**
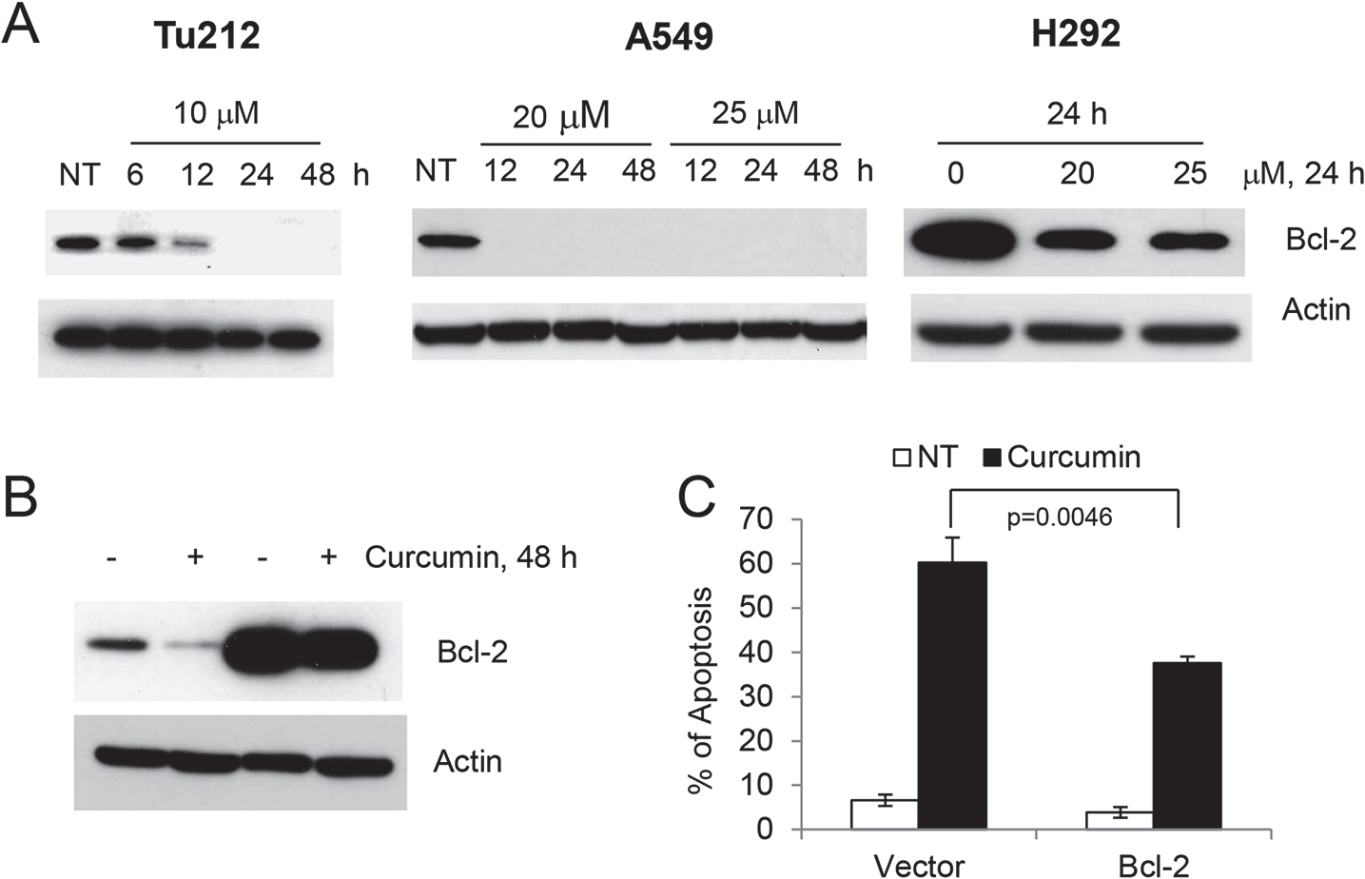
Inhibition of Bcl-2 is required for curcumin-induced apoptosis. Tu212, A549 and H292 cells were treated with curcumin and expression of Bcl-2 was examined by immunoblotting. (B) 041 and 041 Bcl-2 overexpressing cells were treated with 10 μM of curcumin for 48 h and expression of Bcl-2 was measured. (C) 041 and 041 Bcl-2 overexpressing cells were treated with 10 μM of curcumin for 48 h and apoptosis was measured by annexin V staining. Average results from three independent experiments were plotted with standard deviation as error bars. p value was determined by student t-test. p<0.05 was considered statistically significant.

Bcl-2 is one of the most potent anti-apoptotic proteins; it is overexpressed in many types of human tumors and protects cells from mitochondria-mediated apoptosis induced by a wide variety of stimuli, including chemotherapeutic drugs and gamma-irradiation [19]. Since curcumin induced mitochondria-mediated apoptosis as evidenced by the release of cytochrome C in the cytoplasm (Fig 2D), we next examined the expression of Bcl-2 in both SCCHN and lung cancer cell lines. Treatment with curcumin strongly inhibited the expression of Bcl-2 in all cell lines tested (Fig 6A). To confirm that inhibition of Bcl-2 plays a role in apoptosis, we overexpressed Bcl-2 in 041 cells (Fig 6B) and measured apoptosis. As shown in Fig 6C, overexpression of Bcl-2 significantly protected cells from curcumin-induced apoptosis, suggesting that inhibition of Bcl-2 is also required for curcumin-induced apoptosis.

## Discussion

Accumulated evidence suggests that combination approaches to cancer therapy are much more successful and are more commonly utilized than single agent approaches because of their ability to impact multiple pathways. Each component of the combination has different molecular targets and affects different pathways, thus minimizing primary as well as acquired resistance. Considering this fact, natural compounds, also called “dirty compounds” because of their undefined and wide/numerous molecular targets, are well suited for the purpose of chemoprevention and therapy. Most natural compounds context-dependently affect multiple signaling pathways [2]. On the other hand, induction of apoptosis is the key for successful tumor regression or elimination of abnormal premalignant cells. Other anti-proliferative effects such as cell cycle arrest may result in stable disease at best. In the current study, we observed that the natural compound curcumin dose-dependently induced apoptosis in most SCCHN and lung cancer cells lines as evidenced by Annexin V staining, activation of caspase 3 and cleavage of PARP. We further defined that curcumin-induced apoptosis is mediated by modulation of multiple molecular targets such as p73, AKT and Bcl-2. We also demonstrated that the apoptosis is independent of p53.

The tumor suppressor protein p53 plays a crucial role in apoptosis and patients with wild-type p53 have better outcome from chemotherapy than those with deleted or mutated 53 [20]. Using isogenic cell lines or p53 siRNA, several studies have clearly demonstrated that curcumin increased the expression of p53 and induced p53-dependent apoptosis [21, 22], although curcumin also induced apoptosis in cells expressing mutant or no p53. Our findings clearly demonstrate that although curcumin increased p53 protein levels, p53 has no significant role in apoptosis induction by curcumin in lung cancer cell lines. Knock down of p53 had no impact on cellular growth in two different lung cancer cell lines. On the other hand, curcumin induced apoptosis of other cell lines that express mutant p53 (Tu212) or no p53 (H1299, 886LN). Unlike p53, the family member p73 is frequently functional in cancers and plays important roles in determining cellular sensitivity to many anti-cancer drugs [23, 24]. In search for the mechanism of curcumin-induced apoptosis, we found that curcumin treatment also induced the expression of p73, mainly the β-isoform and that inactivation of p73 protected cells from curcumin-induced apoptosis in two different cell lines. To our knowledge, this is the first report to show that curcumin increases the expression of p73 and that inactivation of p73 protects cells from curcumin-induced apoptosis, and is consistent with many other studies showing that p73 is sufficient to induce apoptosis [10, 13, 14].

After exposure to cytotoxic drugs, cancer cells may undergo apoptosis either through activation of the death receptor pathway or mitochondrial depolarization. Bcl-2 serves as the mitochondrial gate-keeper, is frequently overexpressed in tumors and plays a critical role in chemo- and radio resistance [19, 25]. The Bax to Bcl-2 ratio is critical for maintaining mitochondrial membrane integrity. Downregulation of Bcl-2, even without upregulation of Bax, shifts the ratio towards apoptosis. siRNA-mediated downregulation or small molecule inhibition of Bcl-2 are sufficient to induce apoptosis in cells that are dependent on Bcl-2 [26–28]. We found that curcumin strongly inhibited the expression of Bcl-2 in all cell lines tested. Moreover, overexpression of Bcl-2 protected cells from curcumin-induced apoptosis. These results suggest that curcumin-induced apoptosis is also mediated via downregulation of anti-apoptotic Bcl-2 protein.

Finally, tobacco-related malignancies, mainly represented by lung cancer and SCCHN, result from the activation of oncogenic pathways such as EGFR, K-RAS and H-RAS, PI3K-CA etc. The AKT serine-threonine kinases are common downstream effectors of these oncogenic pathways and are critical for tumorigenesis [29, 30]. It was previously reported that curcumin inhibited phosphorylated AKT in many cancer types. Consistent with these reports, we also found that curcumin strongly inhibited phosphorylated AKT in tobacco-related cancer cell lines. Interestingly, rescue of phosphorylated AKT through overexpression of CA-AKT significantly protected cells, suggesting the critical role of p-AKT inhibition in curcumin-induced apoptosis. In conclusion, our study identifies the natural compound curcumin as a multi-targeted agent. It simultaneously activates the tumor suppressor p53/p73 pathways and inhibits pro-apoptotic p-AKT and Bcl-2, thus strongly inducing apoptosis in tobacco carcinogen-induced cancer cell lines.

## Supporting Information

S1 Fig Dose dependent growth inhibition of SCCHN cell lines by curcumin. Cells were seeded in 6-well plates at 30–40% confluency and treated with the indicated concentrations of curcumin after overnight incubation. Plates were stained with methylene blue after 7 days.
(TIF)
