# Supplementary figures and images for: Curcumin Induces Apoptosis of Upper Aerodigestive Tract Cancer Cells by Targeting Multiple Pathways
Source: PLoS One. 2015 Apr 24;10(4):e0124218. doi: 10.1371/journal.pone.0124218 (PMC4409383; doi:10.1371/journal.pone.0124218)

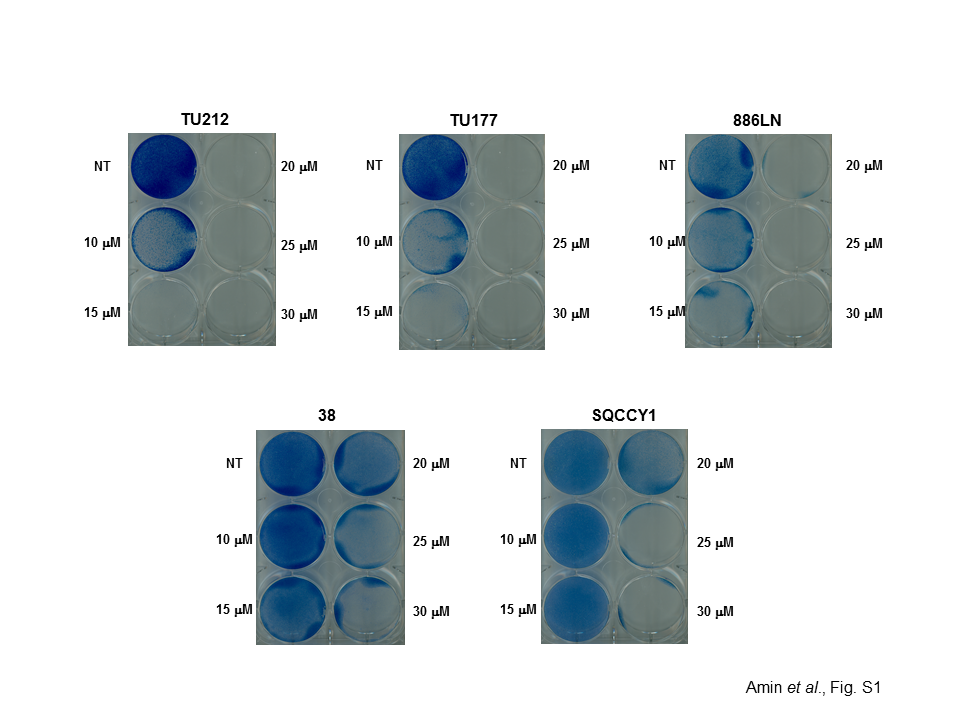

Supplement: S1 Fig — Cells were seeded in 6-well plates at 30–40% confluency and treated with the indicated concentrations of curcumin after overnight incubation. Plates were stained with methylene blue after 7 days. (TIF) [file pone.0124218.s001.tif]
